# Traditional and cortical trajectory screws of static and dynamic lumbar fixation- a finite element study

**DOI:** 10.1186/s12891-020-03437-5

**Published:** 2020-07-14

**Authors:** Che-Wei Liu, Lu-Lin Wang, Yu-Kun Xu, Chun-Ming Chen, Jian-Cyuan Wang, Wei-Tsung Tsai, Shang-Chih Lin

**Affiliations:** 1 Graduate Institute of Applied Science and Technology, National Taiwan University of Science and Technology, Taipei, Taiwan; 2 Department of Orthopaedics, Cathay General Hospital, Taipei, Taiwan; 3 Department of Orthopaedics, Zhangzhou Affiliated Hospital of Fujian Medical University, Zhangzhou, China; 4 Graduate Institute of Biomedical Engineering, National Taiwan University of Science and Technology, No. 43, Sec. 4, Keelung Rd, Taipei, 106 Taiwan, Republic of China

**Keywords:** Pedicle, Cortical bone trajectory, Lumbar instability, Adjacent segment degeneration, Finite-element analysis

## Abstract

**Background:**

Two types of screw trajectories are commonly used in lumbar surgery. Both traditional trajectory (TT) and cortical bone trajectory (CBT) were shown to provide equivalent pull-out strengths of a screw. CBT utilizing a laterally-directed trajectory engaging only cortical bone in the pedicle is widely used in minimal invasive spine posterior fusion surgery. It has been demonstrated that CBT exerts a lower likelihood of violating the facet joint, and superior pull-out strength than the TT screws, especially in osteoporotic vertebral body. No design yet to apply this trajectory to dynamic fixation. To evaluate kinetic and kinematic behavior in both static and dynamic CBT fixation a finite element study was designed. This study aimed to simulate the biomechanics of CBT-based dynamic system for an evaluation of CBT dynamization.

**Methods:**

A validated nonlinearly lumbosacral finite-element model was used to simulate four variations of screw fixation. Responses of both implant (screw stress) and tissues (disc motion, disc stress, and facet force) at the upper adjacent (L3-L4) and fixed (L4-L5) segments were used as the evaluation indices. Flexion, extension, bending, and rotation of both TT and CBT screws were simulated in this study for comparison.

**Results:**

The results showed that the TT static was the most effective stabilizer to the L4-L5 segment, followed by CBT static, TT dynamic, and the CBT dynamic, which was the least effective. Dynamization of the TT and CBT fixators decreased stability of the fixed segment and alleviate adjacent segment stress compensation. The 3.5-mm diameter CBT screw deteriorated stress distribution and rendered it vulnerable to bone-screw loosening and fatigue cracking.

**Conclusions:**

Modeling the effects of TT and CBT fixation in a full lumbosacral model suggest that dynamic TT provide slightly superior stability compared with dynamic CBT especially in bending and rotation. In dynamic CBT design, large diameter screws might avoid issues with loosening and cracking.

## Background

Fusion surgeries are commonly employed for the treatment of spinal pathologies such as spondylolisthesis, degenerative diseases, trauma, and neoplasms. Transpedicular fixation through interconnected rods remains the “gold standard” in fusion surgery [1, 2]. The most commonly adopted transpedicular fixation is the traditional trajectory (TT) of the pedicle screw, which follows the anatomic axis of the pedicle into the cancellous bone of the vertebral body. Various factors affect the pull-out strength of the TT screws such as screw design, trajectory, and insertion point, etc. [3, 4] Convergent trajectory of TT screw increases pull-out strength by 28.6% compared with a straight-in screw [3]. Another approach to increase pull-out strength is to possess a more medialized starting point which is closer to the cortical pars interarticularis with vertical trajectory [4].

Cortical bone trajectory (CBT) is a recently developed technique which utilizes a laterally-directed trajectory that follows a caudocephalad path sagittal and a laterally-directed path in the transverse plane, engaging only cortical bone in the pedicle without involvement of the vertebral body trabecular space [5]. Santoni et al. [5] proposed a shorter and slimmer screw design whose specific trajectory allowed maximization of contact with the cortical bone. It has been demonstrated that CBT exerts a lower likelihood of violating the facet joint [6], and superior pull-out strength than the TT screws, especially in osteoporotic vertebral body [5–9]. Additionally, due to its diverging trajectory, CBT results in minimal surgical wound and multifidus muscle destruction [10]. Overall, there is evidence for CBT to exert similar or reduced postoperative pain and blood loss compared to the TT technique [11–13]. The decrease in postoperative bed-time was attributed to the smaller incision size, decreased disruption of muscle attachment, and soft-tissue dissection using CBT [14].

Varieties of spinal dynamic fixators have been used such as Cosmic or Dynesys systems (pedicle screw implants) and DIAM and Corflex (interspinous process implant). Among them, the Dynesys system is the most widely-used dynamic fixator that prevents adjacent segment disease (ASD) progression and provides support for fusion surgery [15–17]. Recently, a top-loading and minimally invasive Dynesys system was designed for surgery, but performed poorly when implanted in the osteoporotic bone; the tendency of the pedicle screw trajectory to violate the superior facet joint which may lead to ASD progression [18–20].

The CBT screws with 3.5 mm to 5.5 mm diameter were commonly employed in clinical practice. While most studies empathize the equivalent biomechanical strength between TT and CBT, those conclusions are drawn based on 5.5 mm (and above) diameter CBT screws [5, 7, 8, 21]. This study aimed to investigate the biomechanics of the slim CBT-based static and dynamic systems for evaluating the advantages and disadvantages of applying CBT for dynamic fixation. We assume that CBT may work as an alternative fixation base for dynamic fixation, especially for osteoporotic spine.

## Methods

### Lumbar column from L1 to S1 levels

Based on CT-scanning images, a baseline comprising the lumbosacral column with healthy segments from L1 to S1 levels was identified in the authors’ laboratory. The nonparallel gaps of the facet surfaces consistently measured approximately 0.5 mm in the unloaded neutral position. The material properties of all implants and lumbosacral tissues were adapted from a previous model of the current authors that has been validated from cadaveric and numerical data [22].

### Static and dynamic traditional and cortical constructs

**Fig. 1:**
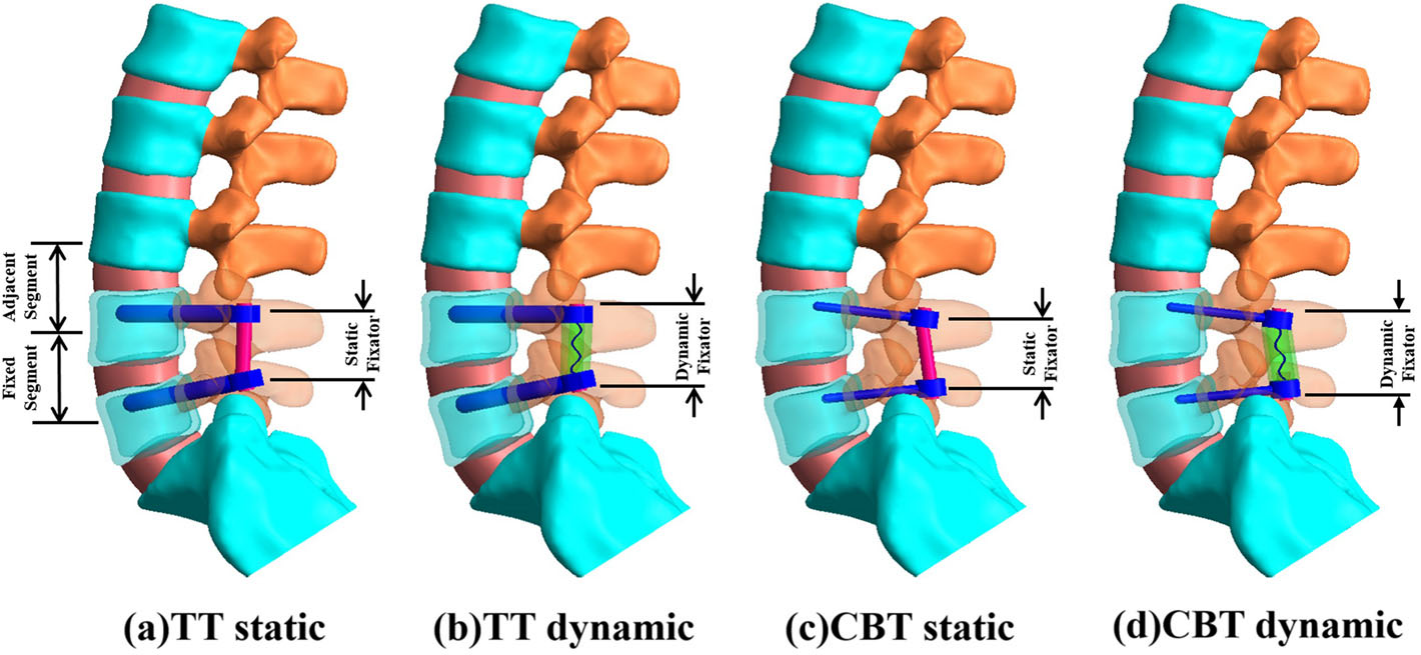
Four highly nonlinear lumbosacral models used in this study. TT static fixation(a). TT dynamic fixation(b). CBT static fixation(c). CBT dynamic fixation(d). The adjacent (L3-L4) and fixed (L4-L5) segments are chosen as the representatives of the tissue responses

**Fig. 2:**
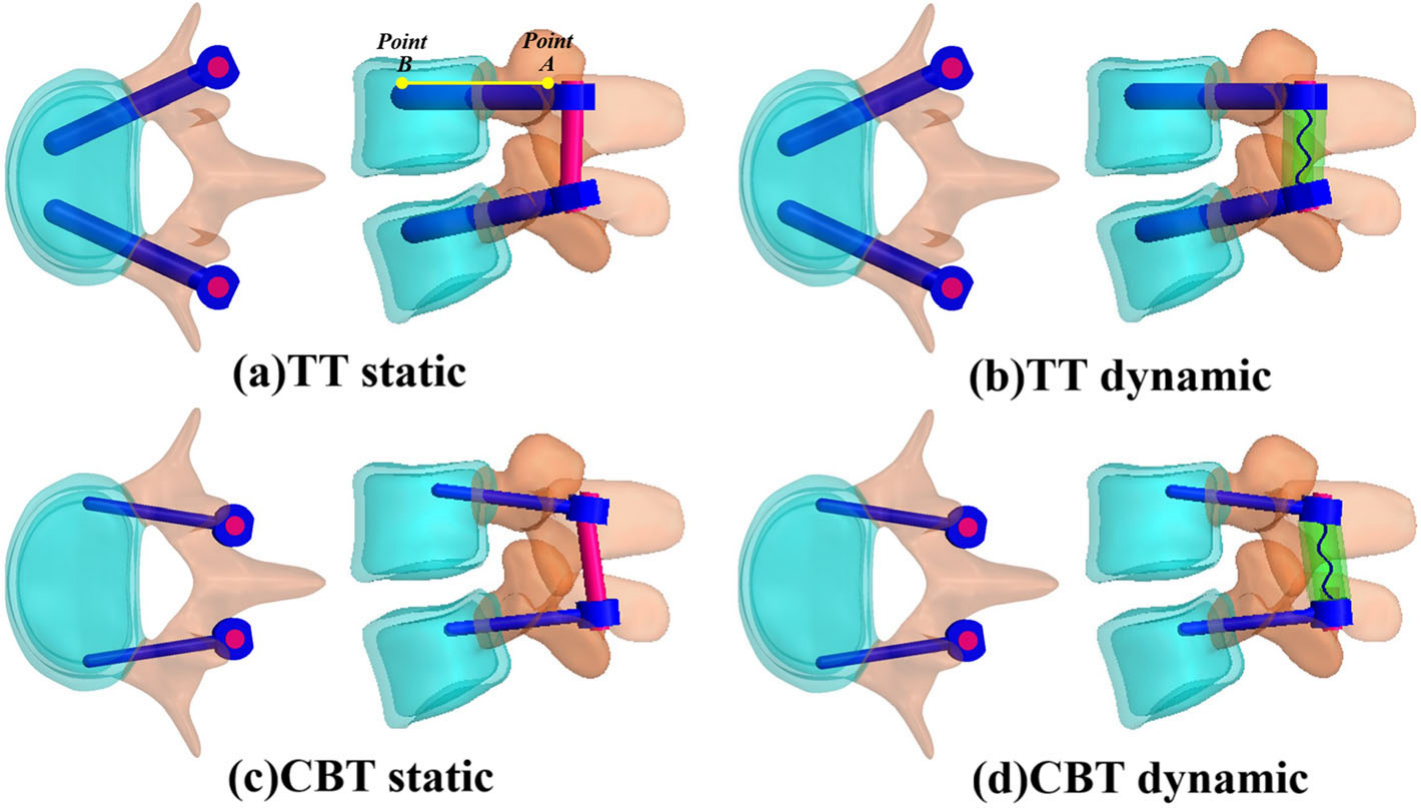
Four diagrams to illustrate the trajectory of screws from top and side views. TT static fixation(a). TT dynamic fixation(b). CBT static fixation(c). CBT dynamic fixation(d). The definitions of the *Line AB* and the *Points A*, and B are described in the content

Four variations of screw constructs were included in this study: TT static, TT dynamic, CBT static, and CBT dynamic models. These were instrumented into the healthy L4-L5 segments. Two static and dynamic TT fixators were used to immobilize the L4-L5 segment to serve as the comparison baselines (Fig. 1a, b, Fig. 2a, and b). As counterparts, the static and dynamic CBT fixators were instrumented at the same levels. The entry point of the CBT technique screw was located in the lateral point of the pars interarticularis projecting in the 5-o’clock orientation in the left pedicle and the 7-o’clock orientation in the right pedicle, using the face of a clock orientation. CBT screws were inserted 10° laterally in the axial plane and 25° cranially in the sagittal plane [5, 8, 9]. (Fig. 1c, d, Fig. 2c, and d). For the sake of equivalent comparisons, the two static fixators were titanium-based rods consistently measuring 5.5-mm in diameter. TT and CBT screws measure 5.5- and 3.5-mm in diameter, respectively. The simulative mechanism of the TT and CBT dynamic fixator were previously described as space and cord Dynesys like construct [22]. Between static and dynamic fixations, the insertion site orientations of the pedicle screws and the rod curvature were modeled under the guidance of an orthopedic surgeon. The top- and side-view trajectories of the TT and CBT screws are shown in Fig. 2. *Line AB* denotes the distribution of stress along the screw shaft (Fig. 2a). *Points A* and *B* are located at the screw tip and hub (junction between the smooth and threaded regions) respectively.

The configurations of all implant components were developed using the SolidWorks, Ed. 2018 (SolidWorks Corporation, Concord, MA, USA) software. The screw threads are excluded for simulation since the bone-screw slippage was not a major concern in this study. The metallic components of the fixators were made from a titanium-based alloy (Ti-6Al-4 V ELI). An assumption of linear elasticity was assigned for all implant materials and further validated by comparing the calculated von Mises stresses with the yielding strength of the corresponding material.

### Finite element analyses and validation

The lumbosacral model was fixed at the S1 bottom and loaded at the L1 top to activate flexion, extension, right bending, and right rotation. The interfaces of the facet joints were modeled as surface-to-surface contact elements, which allowed separation and slippage. The articulating friction was ignored and only transmitted normal forces were considered. The interfaces of other tissues were assumed bonded. The bone-screw interfaces were modeled as surface-to-surface contact elements to calculate the contact force between the Line AB (Fig. 2a) [22]. An automatic mesh generation algorithm was used with the software Simulation Ed. 2018 (SolidWorks Corporation, Concord, MA, USA). The meshing strategy was designed for curved element boundary, and thus, avoided sharp discontinuities to induce an unrealistically high-stress concentration. Using the aspect ratio and Jacobian checks, all elements were within acceptable distortion limits to maximize the accuracy of our results. The models were meshed by the ten-node tetrahedral solid elements. On average, the final meshes of the three lumbosacral models consisted of about 231,000 elements and 340,000 nodes. Mesh refinement was performed for modeling accuracy until excellent monotonic convergence behavior with < 5% difference in the total strain energy was achieved. The nonlinear algorithm with a large-deformation formula and direct-sparse solver was used in the software Simulation Ed. 2018 (SolidWorks Corporation, Concord, MA, USA).

The criterion for controlling the values of entire lumbosacral motion was adapted as a reasonable approach to evaluate implant-induced effects on the adjacent segments [11, 14, 15]. The applied displacement onto the L1 vertebra ensured the disc ROMs of the adjacent, transition, and fixation segments of the healthy model comparable to the cadaveric data obtained from the study by Yamamoto et al. [23] The disk ROM was defined as the difference between disk angles before and after lumbar motion. The nonlinear algorithm with a large-deformation formula and direct-sparse solver was used in the software Simulation Ed. 2018 (SolidWorks Corporation, Concord, MA, USA).

Four indices were chosen to evaluate the trajectory- and dynamization-related effects of the TT and CBT screws on tissue responses and fixator behaviors. Tissue responses at the fixed and adjacent segments were evaluated in terms of disk ROM, disc stress and facet force. The stress distribution at the bone-screw interfaces was used as an index for fixator behavior. The stress distribution along the *Line AB* was denoted as the potential of loosening failure for the pedicle screw (Fig. 2a). The von Mises stress was chosen as the equivalent stresses of discs and screws in this study.

## Results

### Disc ROMs

**Fig. 3:**
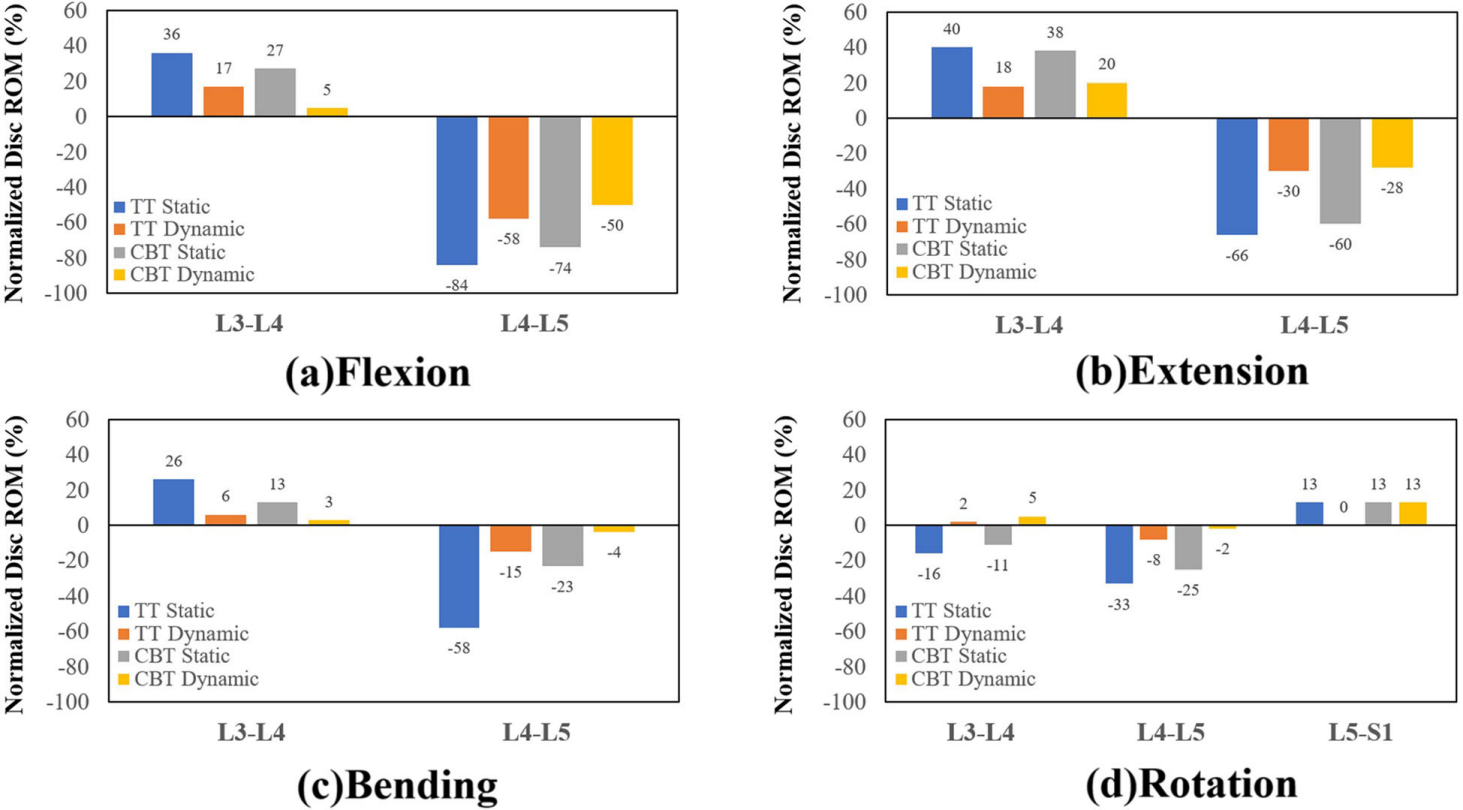
Normalized disc ROMs of the different fixators at the adjacent and fixed segments in four motions Flexion(a). Extension(b). Bending(c). Rotation(d).

Except for rotation, all fixations consistently showed a significant motion reduction of the fixed disc and motion compensation in adjacent discs for all motions (Fig. 3). As an effective stabilizer to the L4-L5 segment, the TT static performed the best, followed by the CBT static, TT dynamic, and the CBT dynamic. For bending and rotation, the normalized disc ROMs reduction of the CBT dynamic was less than − 5% (Fig. 3). These findings indicate the potentially insufficient stability provided by the CBT dynamic to the fixed segment in bending and rotation. Subsequently, the increase in disc ROM induced at the adjacent segment was highest for the TT static.

### Disc stresses

**Fig. 4:**
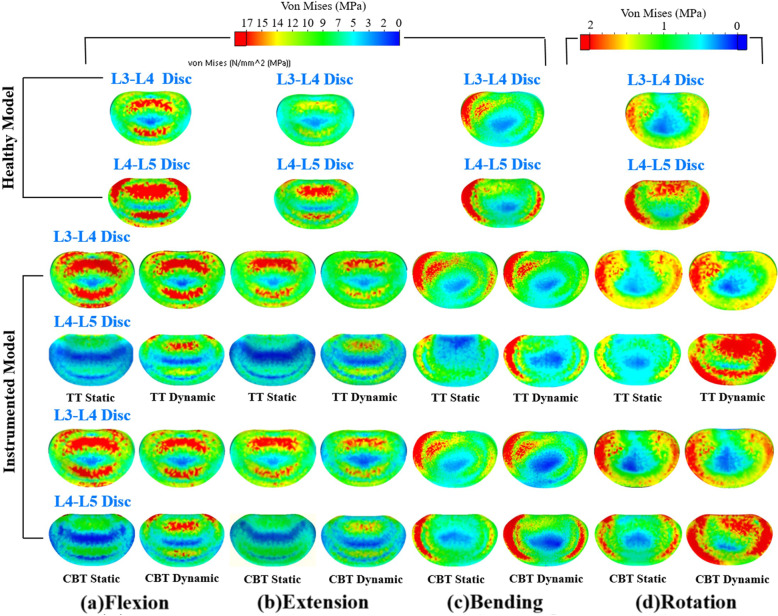
Stress contours over L3-L4 (upper) and L4-L5 (lower) discs of the different fixators in four motions. The upper and lower are the healthy and instrumented models, respectively. The stress scales are the same except for rotation  Flexion(a). Extension(b). Bending(c). Rotation(d).

**Fig. 5:**
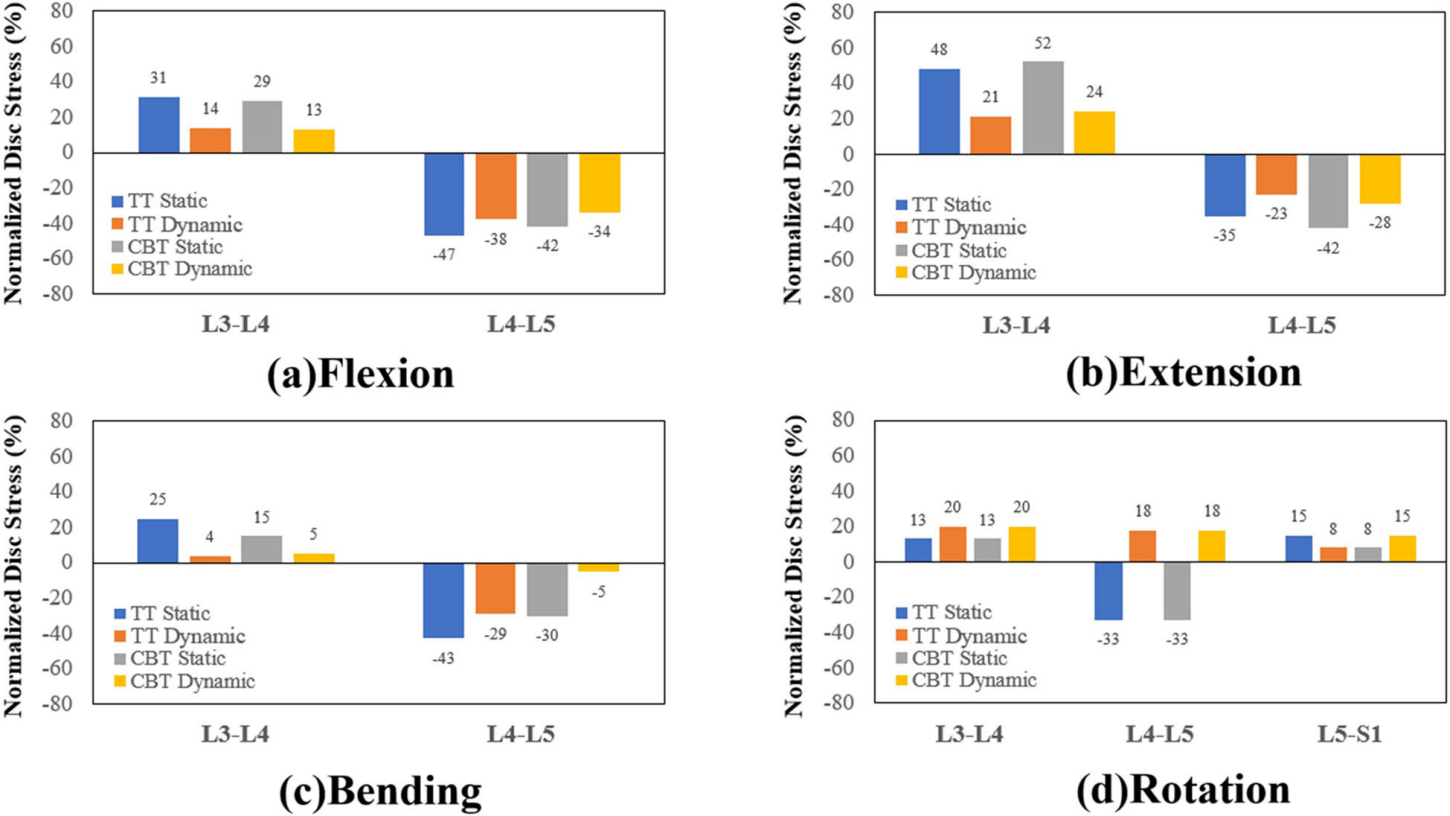
Normalized disc stress of the different fixators at the adjacent and fixed segments in four motions Flexion(a). Extension(b). Bending(c). Rotation(d).

For the healthy and instrumented models, the stress-distributing contours of the adjacent and fixed discs can provide a visual comparison of the stabilizing ability and adjacent segment compensation among four fixations (Fig. 4). Similar to the disc ROMs, the stiffer TT static showed the least and the most stressing contours at the L4-L5 and L3-L4 discs, respectively, than the others. Consistently, the lighter stress contours of dynamic fixation showed reduced load-bearing ability of the screw-spacer construct for the TT and CBT fixators. The reddish stress contours at the L3-L4 disc correlated well to the kinematic compensation from the fixed to adjacent discs. Among the fixators, quantitative comparisons between the disc stresses are shown in Fig. 5. Except in lateral bending, the static TT and CBT fixators induced nearly equivalent increases in the L3-L4 disc stress. Except for rotation, the L3-L4 disc stresses of the TT dynamic and CBT dynamic were significantly reduced compared to their counterparts. Dynamization did not suppress the rotational adjacent segment stress compensation at the L3-L4 disc (Fig. 4d).

Except for rotation, the TT static provided the highest load-sharing ability to the fixed segment, followed by the CBT static, the TT dynamic, and the CBT dynamic, which showed the least ability. The CBT dynamic behaved as a compatible stabilizer to the TT dynamic for flexion and extension and provided a minor stabilizing ability to the fixed segment for bending (Fig. 4c). Interesting, the static TT and CBT provided a negative adjacent segment compensation subjected to rotational motion (Fig. 4d). The rotational behaviors of the static TT and CBT were described in the Discussion section.

### Facet forces

**Fig. 6:**
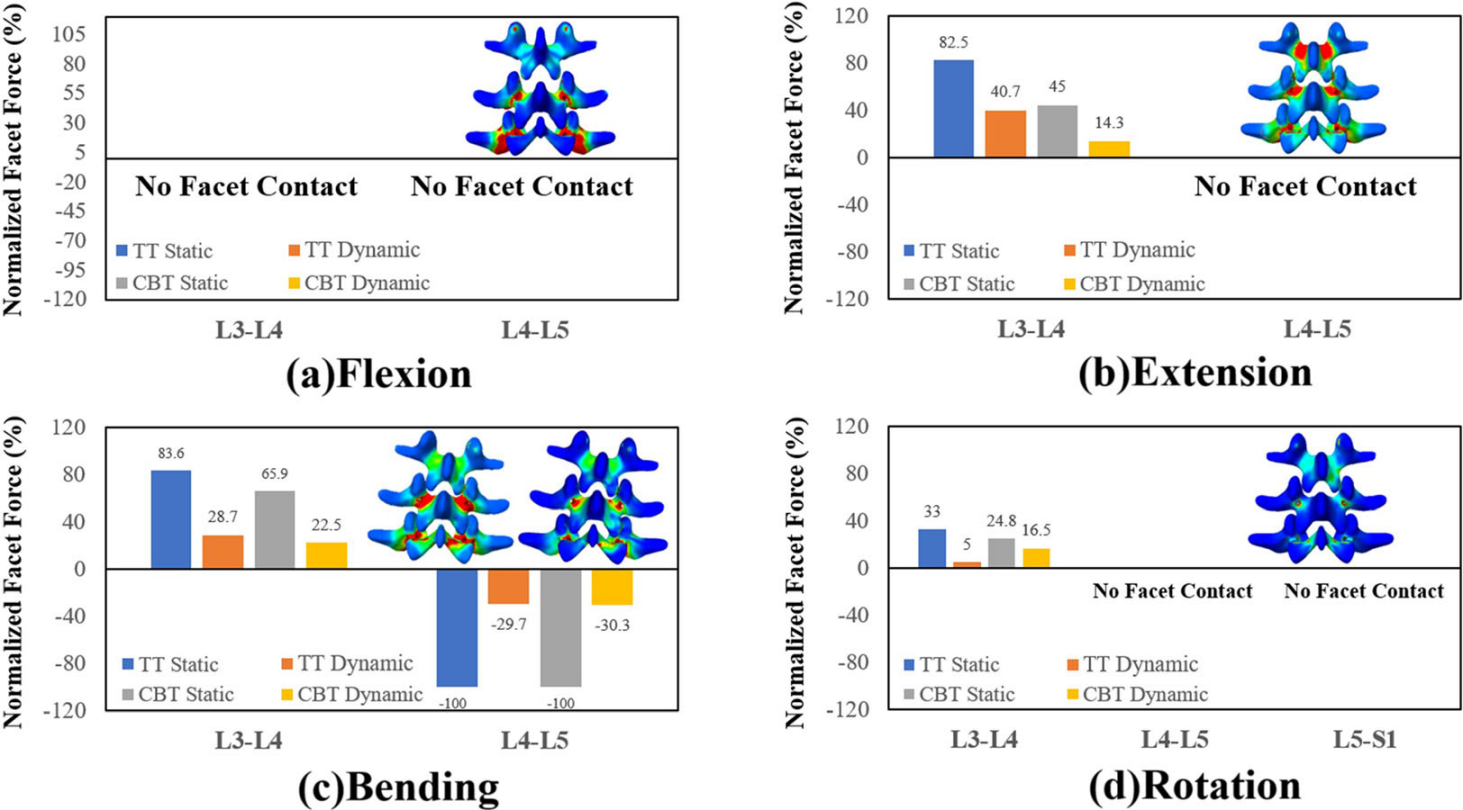
Normalized facet force of the different fixators at the adjacent and fixed segments in four motions. Flexion(a). Extension(b). Bending(c). Rotation(d).

The flexion rendered the paired facet prone to separation and facet contact did not occur at the L3-L4 and L4-L5 segments (Fig. 6a). For extension and bending, kinetic compensation at the L3-L4 facet force was the most remarkable for TT static, followed by CBT static, TT dynamic, and CBT dynamic, which was the least (Fig. 6b and c). No interfacial contact at the L4-L5 facet joints occurred for bending and rotation (Fig. 6c and d). Due to the flexibility of the Dynesys spacer, the dynamization of the TT and CBT fixators allowed the paired L4-L5 facet joints to make contact and thereby reduce the facet forces (Fig. 6c).

### Screw stresses

**Fig. 7:**
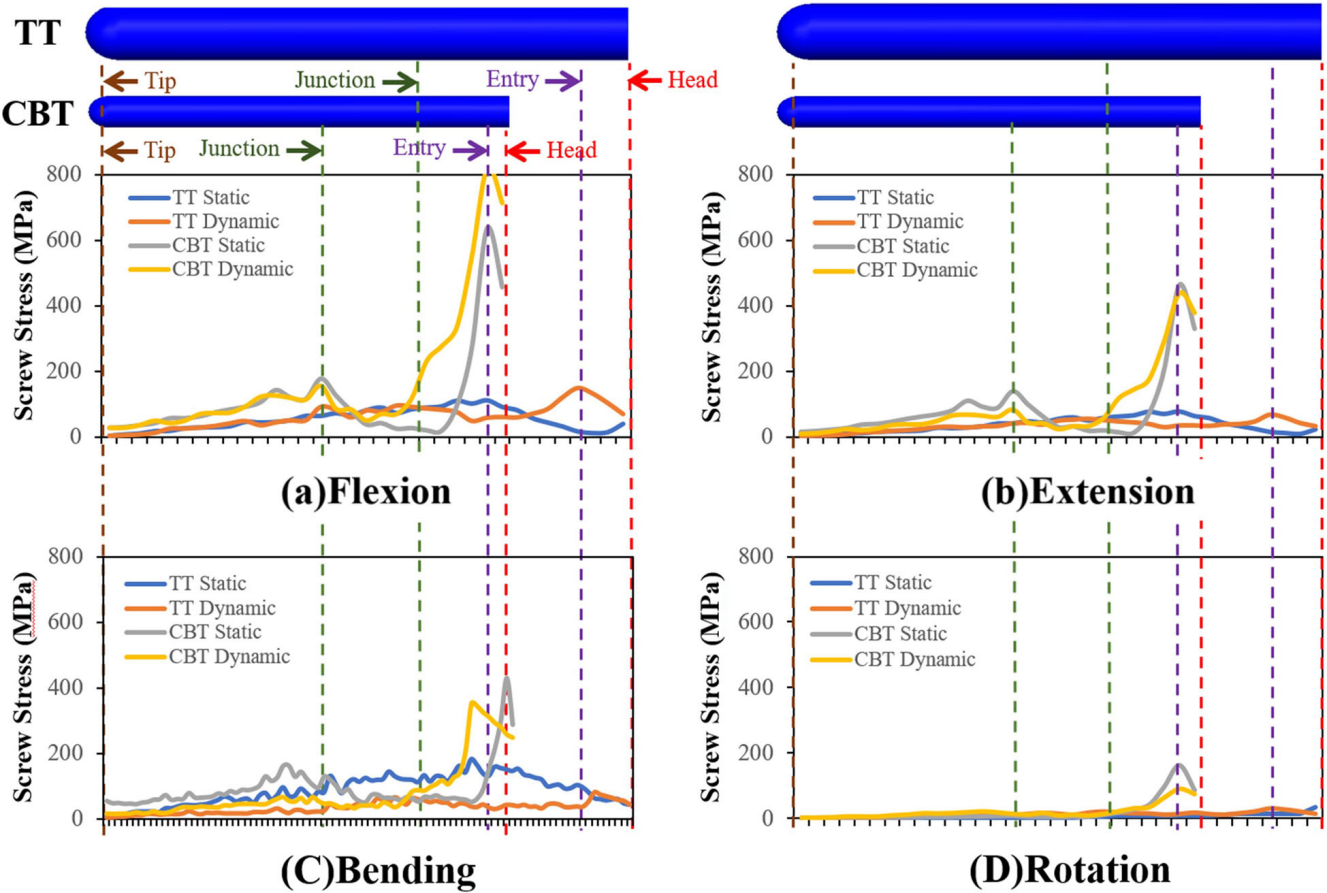
The distribution of screw stress of the different fixators along the bone-screw interfaces (*Line AB*). The terms “Head”, “Entry”, “Junction”, and “Tip” denote screw head (i.e. screw hub), entry site of pedicle screw into posterior element, interface between posterior element and cortical bone, and screw tip. Flexion(a). Extension(b). Bending(c). Rotation(d).

The withdrawal possibility between the bone and screw was measured by the nodal stresses along the *Line AB* (Fig. 2). Two special sites, screw-bone entry, and cortex-cancellous junction were marked as the boundary and material discontinuities. Two types of TT and CBT screws were aligned at the tips for clarity and were subjected to various stress distribution (Fig. 7). In general, all fixators showed high stress at the entry sites, followed by the junction sites. Regardless of the static and dynamic fixator used, the slim CBT screws were highly stressed compared to their counterparts for all motion, which indicated that the 3.5 mm diameter CBT screw had a higher propensity to fail due to loosening and even cracking than the 5.5 mm diameter TT screw.

## Discussion

From the biomechanical viewpoint, four screws and two longitudinal rods form a three-dimensional construct to stabilize the instrumented segment. The two trajectories of screw insertion result in two biomechanical responses: the bony contact of posterior element, cancellous core, and cortical shell along the screw length and the stabilizing base that is spanned by the paired screws. The two types of TT and CBT screws showed distinct modes of bone contact. Significant variations in screw pull-out strength and the higher purchasing ability of the 5.5 mm diameter CBT than TT screws were demonstrated previously [5, 7–9], but this was not the case with the 3.5 mm diameter CBT screw stress.

The dynamization of screw fixation aims to provide flexibility to the adjacent segments and suppress the postoperative ASD problem. There was less ROM constraint (− 50% flexion, − 28% extension in CBT dynamic and − 58, − 30% in TT dynamic) and lower stress sharing (− 34, − 28% in CBT dynamic and − 38, − 23% in TT dynamic) in dynamic CBT in flexion and extension. Similar to previous findings in bending, there was weaker fixation strength in bending in both dynamic and static CBT compared with TT (Fig. 3c and Fig. 5c) [5, 7]. Interestingly, in rotation, both static TT and CBT showed a 33% reduction in normalized disc stress. In dynamic TT and CBT, however, the disc stress adversely increased by 18% (Fig. 5d), which may be attributed to pretension in the cord.

**Fig. 8:**
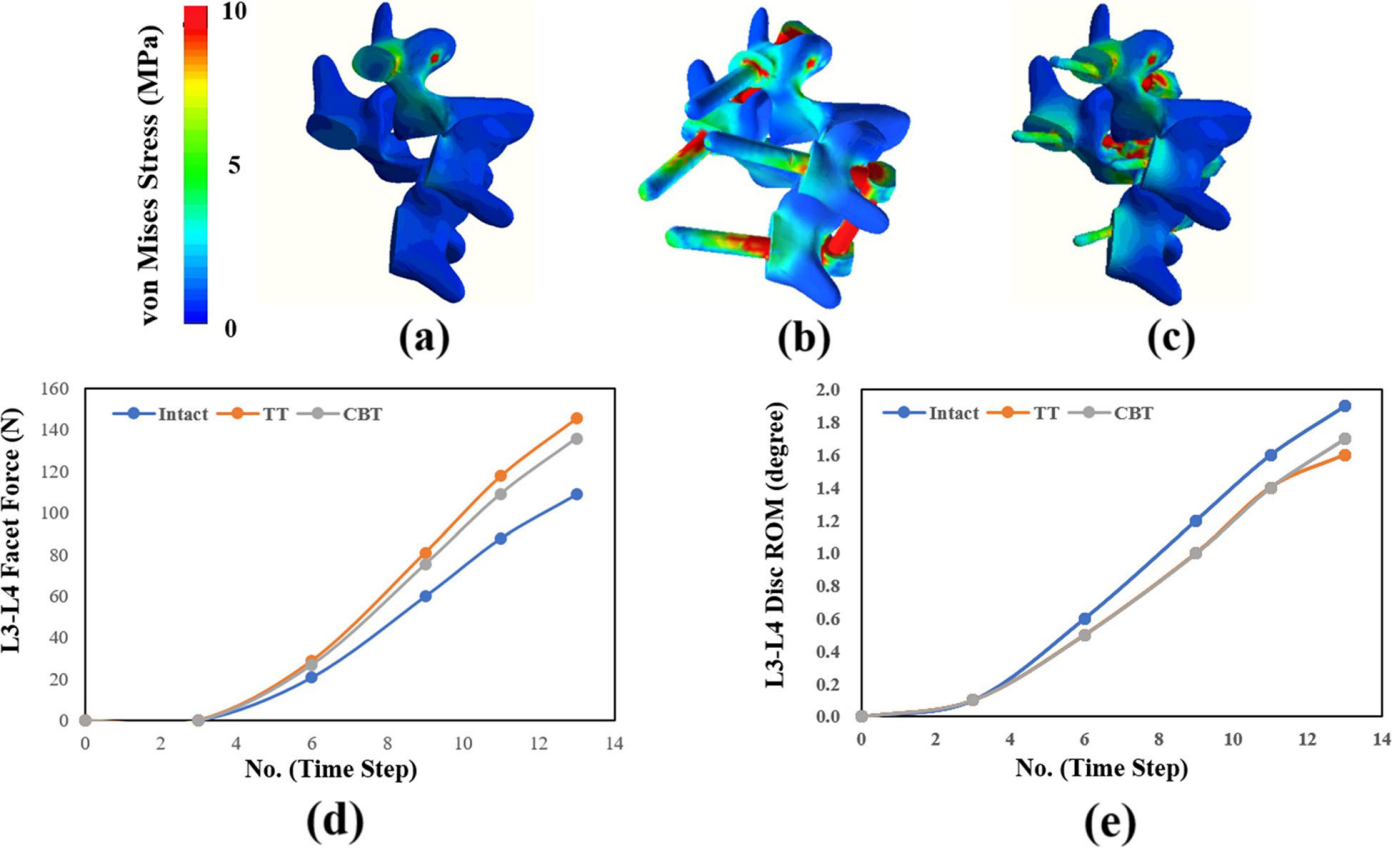
The earlier contact of the L3-l4 facet joints was used to account for the negative adjacent segment compensation of the static TT and CBT in rotation. **a**, **b**, and **c** show the facet contact for the intact, TT, and CBT models, respectively. **d** and **e** show the increased facet forces and disc ROMs of the L3-L4 segment in terms of time step no. that indicates the execution sequence of the finite-element analysis

The entire lumbosacral model provides more detailed information about biomechanical behaviors of fixed and adjacent segments. Interesting, the adjacent segment compensation in rotation is different from the other motion (Fig. 3d). The rotation ROM did not show compensation in L3-L4 after static TT and CBT fixation. This research accounted for this behavior by comparing facet force and disc ROM in intact and static TT and CBT models in the time curve of the finite-element analysis (Fig. 8). For static TT and CBT, the faster increase in L3-L4 facet force shows earlier contact of the paired facet after fixation and thus deteriorates the kinetic and kinematic compensation for rotation.

Among four fixation methods, the TT static behaved as a more constrained stabilizer to the fixed segments. These surgery-related findings indicate that the choice of the static or dynamic fixators is potentially dependent on the stability demand of the fixed segment and degeneration degree of the adjacent segments. If the structural integrity of the fixed segment is not the first requirement, the CBT dynamic might be a recommended option in a situation of mild or moderate degeneration at the adjacent segments. However, a static CBT and even a static TT fixation might be adopted if the adjacent segments are still healthy and the fixed segment requires stabilization.

The stress distribution for all fixations showed that stress was concentrated near the screw hub, at the junction of the threaded and unthreaded regions, corroborating reports showing sites with most failure on the TT screws [24]. Interestingly, the static and dynamic CBT fixations consistently showed higher von Mises stress distribution than their counterparts, which indicated that the use of the CBT screws was more prone to screw-bone interface failure. However, the results of loosening the CBT screws were in contrast to previous studies, which revealed compatible or even better pull-out strength and toggle strength of the CBT than TT [7–9]. The simulation in this study showed that the different geometry and mode of bone contact in CBT may subject it to tremendous regional stress, resulting in loosening (bone contact failure) or breakage (screw fatigue). This warrants the need for additional investigation on interface failure using data on screw threads and bone destruction.

There are numerous reports in the literature that attest to the superiority of the CBT screws over TTs for fixation of osteoporotic bone [3, 5–9]. For our study, we divided the vertebral body into three zones, the cancellous core, cortical shell, and posterior element, which correspond to the Young’s modulus of three distinct vertebral components. The TT screw was within the core and the CBT screw served to anchor the cortical shell (Fig. 2). Due to the extreme nonlinearity of the entire lumbosacral column (L1-S1), this study did not use micro-computerized tomography [7] to evaluate the trabecular bone, as our intent was to avoid too complex a model, which inevitably leads to divergence when performing finite-element analysis. Consequently, osteoporosis within the cancellous core was not simulated because the capacity of TT fixation for immobilization (e.g.*,* stability and holding power) was not reduced. Consequently, the predicted results that were based on the assumption of healthy bone might overestimate the capacity of the TT screw to a greater extent than the CBT screw. The biomechanical impact of the osteoporotic vertebral cores will be investigated in future studies.

In clinical practice, adjacent disks are prone to mild or even moderate degeneration; this is observed even when the condition does not require instrumentation. The morphological and structural changes in the adjacent disks in this case make them stiffer than the healthy ones. This had been simulated and described in our previous report [22, 25]. In this study, we assumed that the adjacent disks were healthy so as to evaluate the dynamic effects and trajectories of the TT and CBT screws with respect to the adjacent disks. A stiffer disk can suppress transferred kinematic and kinetic changes from the instrumented segment. Consequently, the assumption of the healthy L3/L4 and L5/S1 disks will maximize the implant-induced effects on the non-stiffened adjacent disks.

Previous studies on CBT screws have focused on non-inferior pull-out and toggle strength in direct comparison to TT screws [5, 8, 9, 21]. Osteoporosis may have smaller impact on CBT screws than on TT screws due to the relative cortical trajectories. No research has been published that addresses segmental stability and relative impact on adjacent spinal levels when comparing these two fixation methods. The results in this study showed that static CBT screws provide inferior segmental stability compared with that promoted by TT screws. However, considering its minimal invasiveness, static CBT screws may still be quite useful in short segment fusion after limited spinal decompression or after discectomy. CBT screws may also be used as an alternative fixation method in osteoporotic patients, as the use of larger cage may compensate for the inferior stability [26]. Pars fractures compromise the cortical trajectory of CBT; as such, this condition should be considered a relative contraindication for their use [27].

Dynamic TT simulates the use of Dynesys. Dynesys is the most widely used dynamic fixation method and is based on use of PSs; however, the clinical results generated by this procedure are not fully clear [20]. In our simulation, the dynamic TT provides sound biomechanical profile, decreases disk stress at the instrumented level, and reduces stress compensation in adjacent disc and facet, similar to previous findings [28–31]. However, no significant clinical benefits of Dynesys were reported in both short term and long-term studies [32]. The gap between the biomechanical studies and clinical results may relate to the destruction of muscle during the surgical approach, disruption of facet joint as well as the impact of instrumentation itself that results in deviation of the motion of specific segments away from what is physiologically within normal limits.

Dynamic CBT is a novel design modification from Dynesys. The simulation shows that dynamic CBT results in only slightly inferior segmental stability compared with dynamic TT. The property makes it a minimally invasive alternative to Dynesys for use in short segment stabilization after discectomy and for low-grade spondylolisthesis. This design may improve the clinical results obtained with Dynesys and reduce the incidence of adjacent segment disease (ASD) by the inherently lower chance of facet joint disruption and/or destruction of the posterior musculature. This method may be valuable for osteoporotic patient who needs dynamic fixation. Future biomechanical studies will focus on its effect on physiological motion of the spine.

As with any finite-element analysis, certain assumption-related limitations were inherent in this study. Some of these limitations, including the morphology and material properties of the tissues involved have been discussed previously [22]. However, the pedicle size has substantial impact on the diameter of the inserted screw; this point was not extensively investigated in this study. Only 3.5 mm diameter CBT screws showed the same effect of the slimmest diameter screws currently in clinical use. The biomechanical effects of screw diameter have been considered extensively in the literature. This study focused the use of dynamic fixation methods and comparing different trajectories. CBT, limited by the anatomy of the posterior element, cannot tolerate screws with same diameter as those used in the PS trajectory, which are usually > 6.0 mm in diameter. Thus, this research did not standardize the sizes of the CBT and TT screws. Future research will focus on the effect of different screw diameters on both stability and stress.

This study simulated the TT and CBT screws as monoaxial elements, and as such, the curvatures of the longitudinal rods differed from those used in the clinic (Fig. 2c and d). For the CBT screw, this renders the two-ended surfaces of the Dynesys spacer as non-orthogonal to the spacer axis. Consequently, the simulation of the dynamic CBT fixation might reflect the actual conditions at the screw-spacer interfaces. In practice, a poly-axial screw can avoid excessive rod curvature and simplifying spacer ends, a point that was not considered in the current study. Due to the high nonlinearity of the entire lumbosacral column (L1-S1), this study is designed to simulate the impact of fixation on segmental stability and adjacent stress rather than on bone-screw failure (e.g.*,* loosening and breakage). Consequently, the simulation omitted screw threads and assumed bone-screw interfaces as bone to raise computational efficiency and convergence. Additionally, the fusion cages were not instrumented into the fixed segment. Given these limitations, the findings obtained in this study that were designed to predict results of transpedicular fixation, might overestimate the screw stresses and underestimate ASD.

Similar to any finite-element method, certain assumption-related limitations were inherent in this study. Some, such as the morphology and material property of the tissues have been previously discussed [22]. However, the pedicle size substantially affects the diameter of the inserted screw and is not extensively investigated in this study. Only 3.5 mm diameter CBT screws were considered to show the effect of the slimmest diameter screws that may be used in clinical use. Future research will work on the effect of different screw diameters on the construct stability and screw stress.

This study simulated the TT and CBT screws as monoaxial types, such that the curvatures of the longitudinal rods differed from that used in the clinic (Fig. 2c and d). For the CBT screw, this renders the two end surfaces of the Dynesys spacer non-orthogonal to the spacer axis. Consequently, the simulation of the dynamic CBT fixation might reflect the actual condition at the screw-spacer interfaces. In practice, the poly-axial screw can avoid excessive rod contouring or simplifying spacer ends, which was not considered in the current study. Additionally, the fusion cages were not instrumented into the fixed segment. The predicted results of only transpedicular fixation, which was obtained in this study, might overestimate the screw stresses and underestimate ASD.

This study is, after all, a computational model study. The validation may not reflect the real behavior of human tissue. Factors such as osteoporosis and disc degeneration were not considered in this study. Although, the computational model may reflect some biomechanical behaviors of the spine, to apply the results in the complex clinical practice, further biomechanical studies are needed to confirm the results.

## Conclusion

Modeling the effects of TT and CBT fixation in a full lumbosacral model suggest that dynamic TT provide slightly superior stability compared with dynamic CBT especially in bending and rotation. In dynamic CBT design, large diameter screws might avoid issues with loosening and cracking. The results of this finite element study reflected important factors to be considered in further biomechanical study. To be used in clinical practice, the results needed to cautiously interpreted.

## Data Availability

The datasets used and/or analysed during the current study are available from the corresponding author on reasonable request.

## Abbreviations

TT: Traditional trajectory
CBT: Cortical bone trajectory
ASD: Adjacent segment disease
PS: Pedicle screw
